# Mechanical Stretch Induces mTOR Recruitment and Activation at the Phosphatidic Acid-Enriched Macropinosome in Muscle Cell

**DOI:** 10.3389/fcell.2019.00078

**Published:** 2019-05-08

**Authors:** Shan-Shan Lin, Ya-Wen Liu

**Affiliations:** ^1^Institute of Molecular Medicine, College of Medicine, National Taiwan University, Taipei, Taiwan; ^2^Center of Precision Medicine, College of Medicine, National Taiwan University, Taipei, Taiwan

**Keywords:** skeletal muscle, exercise, phospholipase D, membrane tension, mechanotransduction

## Abstract

The mammalian target of rapamycin (mTOR) is an evolutionarily conserved kinase which assembles a signaling network that integrates diverse biochemical and mechanical cues to coordinate cell growth and proliferation. Mechanical load has been well-appreciated to induce mTOR activation that leads to skeletal muscle growth through phospholipase D (PLD) activity and phosphatidic acid (PA) production. While PA produced by PLD1 is critical for mTOR activation upon mitogenic stimulation at the lysosome, it is unclear where PA is produced upon mechanical stimulation in skeletal muscle. Here we report that membrane tension fluctuation induces the formation of PA-enriched macropinosome in mouse C2C12-derived myotube by either mechanical stretch or osmotic shock. The tension oscillation-induced PA is accumulated at the membrane of macropinosome, not the lysosome. Furthermore, mTOR is recruited to the PA-enriched macropinosome, and its downstream signaling is activated. Our findings reveal the underpinning of mechanical activation of mTOR signaling, and more importantly, the stretch-induced PA-macropinosome as a new platform for mTOR activation.

## Introduction

The mammalian target of rapamycin (mTOR) is a Ser/Thr kinase that regulates cell growth and proliferation through phosphorylation of critical effectors in multiple anabolic pathways, including protein synthesis, lipogenesis, and nucleotide biogenesis while inhibiting autophagy (Laplante and Sabatini, 2009). mTOR functions in two distinct complexes, mTORC1 or mTORC2, which phosphorylate different set of substrates to regulate cell growth and survival, respectively (Dancey, 2010; Dufour et al., 2011). Importantly, the activity of both mTOR complexes are regulated by a variety of intra- and extra-cellular signals such as amino acids, mitogens and cell stresses to ensure that cells grow only during favorable conditions. It has been well-established that to achieve the activation of mTORC1, both signals from amino acids and mitogens are needed, which facilitates the recruitment of mTORC1 to the lysosomal membrane via Rag small GTPases and activates mTOR by Rheb GTPase separately (Betz and Hall, 2013).

In addition to those GTPases on the lysosomal membrane, it has been revealed that the enzymatic function of phospholipase D 1 (PLD1) which catalyzes phosphatidylcholine into phosphatidic acid (PA), is also required for mTORC1 activation (Fang et al., 2001; Foster et al., 2014). After mitogen stimulation, PLD1 is recruited to lysosome through mitogen-stimulated phosphatidylinositol 3-phosphate-production on the lysosomal membrane and is activated by mitogen-stimulated small GTPases, such as Arf, Rho or Ras (Foster, 2009; Yoon et al., 2011a, 2015). PA is a direct activator for mTORC1 which enhances the kinase activity of mTORC1 both *in vitro* and in cells (Fang et al., 2001; Yoon et al., 2011b). PA binds directly to the FKBP12 rapamycin binding (FRB) domain of mTOR, competes with the inhibitory proteins Deptor and FRKB38, and allosterically activates the activity of mTORC1 (Yoon et al., 2011b, 2015).

In addition to the mitogen-stimulated mTORC1 activation, Chien and his colleagues reported that PLD activity is also required for mTORC1 signaling in skeletal muscle cells upon cyclic mechanical stretching (Hornberger et al., 2006; Hornberger et al., 2007). There are two isozymes of PLD expressing in skeletal muscle, PLD1 and PLD2, with different subcellular distribution that PLD1 was mainly localized to the cytoskeletal fraction, whereas PLD2 was detected mainly in the membrane fraction (Hornberger et al., 2006). Although the critical role of PA and mTOR signaling in skeletal muscle growth is well-appreciated, several questions remain unanswered: which PLD is responsible for the mechanical activation of mTOR signaling in muscle cell? Will mTORC1 also be recruited to lysosome upon mechanical stimulation?

In this study, we utilized different osmotic buffer treatments or cell stretching to mimic the mechanical load on muscle cells and investigated the distribution of PA and mTOR in C2C12 mouse myoblast-derived myotube. We found that membrane tension fluctuation induces the formation of macropinosome which are enriched with PA and PLD2. Furthermore, mTOR was recruited to the macropinosome together with its activation in myotube. Our results demonstrate that membrane tension fluctuation triggers the formation of PA-enriched macropinosome in muscle cell that functions as a platform for mTOR recruitment and activation.

## Materials and Methods

### Cell Culture and Transfection

Mouse-derived C2C12 myoblasts (American Type Culture Collection, CRL-1772) were cultured in growth medium (GM), DMEM supplemented with 2 mM L-glutamine, 1 mM sodium pyruvate, antibiotics and 10% fetal bovine serum (Gibco). To induce differentiation, C2C12 were seeded onto laminin (Invitrogen)-coated glass-bottom dish (Mattek), laminin-coated BioFlex Culture Plate (Flexcell), or laminin-coated coverslips in GM, grown to 90% confluency, and then switched to differentiation medium (DM), which is the same as GM but with 2% horse serum (Gibco). This time point was considered as day 0 of differentiation. For transfection, cells at 70% confluency were transfected with the desired DNA constructs using Lipofectamine 2000 (Invitrogen), as recommended by the manufacturer.

### Reagents

Tetramethylrhodamine -dextran (D1818) and Lysotracker (L7528) were purchased from Invitrogen. Anti-mTOR (7C10, #2983), anti-p70 (#9202), and anti-P-p70 (#9205) antibodies were from Cell Signaling Technologies, and anti-tubulin antibody (T9026) was from Sigma-Aldrich. FIPI (#3600) was purchased from Tocris Bioscience. PABD-GFP was constructed by amplification of the 51–91 residues of yeast Spo20 and was subcloned into pEGFP-N1. GFP-PLD constructs were gifts from Dr. Do Sik Min, Lact-C2-GFP and PLCδ-PH-GFP were from Prof. Fang-Jen Lee, and Lamp1-mCheery was from Prof. Jean-Chen Kuo.

### Microscopy and Immunofluorescence Staining

For live-cell microscopy, cells transfected with interested DNA constructs were seeded on glass-bottom dish (Mattek) and imaged with Zeiss inverted microscopy Axio Observer Z1 at 37°C with 63×, 1.35-NA oil-immersion objective. To image fixed cells, sample slides were observed with confocal microscopy LSM700 with 63×, 1.35-NA oil-immersion objective (Carl Zeiss, Jena, Germany).

### Macropinocytosis Assay

To monitor macropinocytosis upon osmotic shock (OS) treatment, myotubes were incubated with indicated osmotic buffers containing 1 mg/ml rhodamine-dextran (70,000 MW, Invitrogen). After PBS wash for five times, cells were fixed and imaged with confocal microscopy. To monitor stretch-induced macropinocytosis, myoblasts were seeded on laminin-coated flexcell silicon dish and were induced to differentiate for 5 days after PABD-GFP transfection. Day-5 differentiated myotubes were incubated in DM medium containing 1 mg/ml rhodamine-dextran and subjected to radial stretching of 2 min at 20% stretching and followed by 2 min resting. Samples were then washed with PBS for five times, fixed with 4% formaldehyde, cut from the plates, mounted and imaged with confocal microscopy (Zeiss, LSM700).

### Stretching and Immunofluorescence Staining of Myotube

C2C12 cells seeded on laminin-coated silicon membrane of BioFlex Culture Plate (Flexcell) was transfected with PABD-GFP or Lamp1-RFP, and differentiated into myotube by DM incubation. After 5 days of differentiation, myotube was subjected to static, radial stretching with 20% strain by a Flexcell FX-5000T Tension System for 2 min and followed by resting for another 2 min. Samples were then immediately fixed with 4% formaldehyde for 30 min, cut from the plates, permeabilized with 0.1% saponin. After blocking with 3% BSA and 5% normal donkey serum, cells were stained with the anti-mTOR antibody. Samples were then observed under confocal microscope LSM700 with 63×, 1.35-NA oil-immersion objective (Zeiss, LSM700).

### Statistical Analysis

Quantitative data were expressed as mean ± SD of at least three independent experiments. All data were analyzed with Student’s *t*-test or one-way ANOVA followed by a Fisher’s Least Significant Difference *post hoc* analysis. *p*-Values were indicated as #*p* < 0.1; ^∗^*p* < 0.05; ^∗∗^*p* < 0.01; ^∗∗∗^*p* < 0.001. *p* < 0.05 was considered as statistical significance.

## Results

### Acute Tension Fluctuation Induces the Formation of PA-Enriched Macropinosome

**FIGURE 1:**
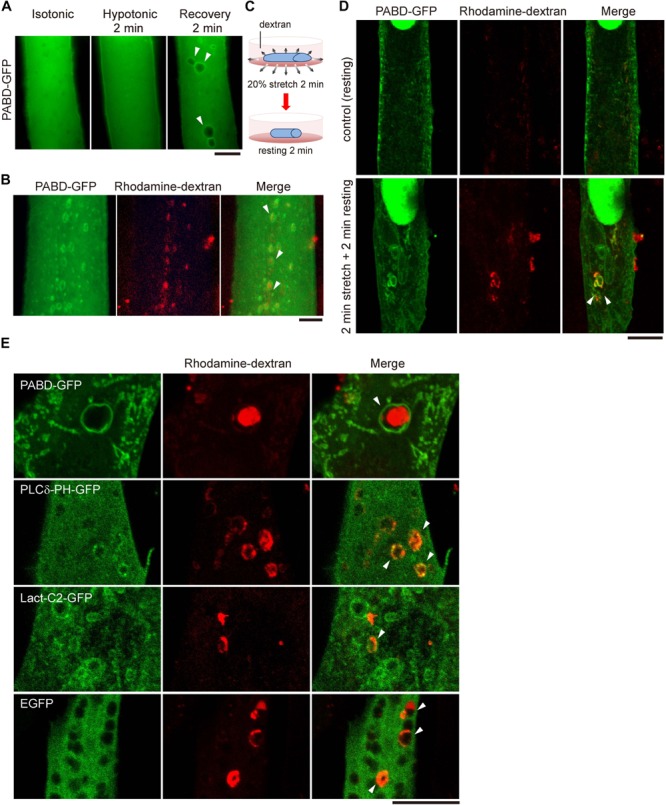
Acute tension fluctuation induces PA-enriched macropinosome formation in myotube. **(A)** Live cell imaging of PABD-GFP expressing myotube. Myotube was imaged first in isotonic buffer (1X PBS), then in hypotonic buffer (0.25X PBS) upon 2-min incubation, subsequently in recovery isotonic buffer (1X PBS) by 2-min interval. Images of the same myotube were acquired by inverted fluorescence microscope. **(B)** OS treatment induced the formation of macropinosome. PABD-GFP expressing myotube was treated with OS in the presence of 1 mg/ml rhodamine-dextran. Image was acquired immediately after PBS wash. **(C,D)** Mechanical stretch induced macropinocytosis in myotube. PABD-GFP expressing myotube cultured on silicon membrane was subjected to 20% radial stretch for 2 min and followed by 2-min resting in the presence of rhodamine-dextran as depicted in **(C)**. After intensive PBS wash, cells were fixed and imaged with confocal microscopy. **(E)** Distribution of lipid biosensors in myotube upon OS treatment. Myotubes expressing control GFP or biosensors for PA (PABD-GFP), PI(4,5)P_2_ (PLCδ-PH-GFP) and PS (Lact-C2-GFP) were subjected to OS in the presence of rhodamine-dextran. Projected confocal images were shown. All arrowheads indicate the PA- and rhodamine-dextran containing macropinosomes. Scale bars: 10 μm.

To explore the distribution of PA in myotube upon membrane tension fluctuation mimicking the muscle stretch and relax, we utilized buffers with different osmolality to induce stepwise tension alteration in myotube. After transfection with a PA biosensor (PABD-GFP, PA-binding domain of yeast Spo20; Zeniou-Meyer et al., 2007), C2C12 mouse myoblast was induced to differentiate with differentiation medium (DM). After 4 days of differentiation, PABD-GFP expressing myotube was imaged with inverted fluorescence microscopy firstly in isotonic buffer (1X PBS), followed by 2-min hypotonic buffer (0.25X PBS) incubation and another 2-min isotonic buffer (1X PBS) recovery (Figure 1A). We hereafter called this treatment of 2-min hypotonic buffer followed by 2-min isotonic buffer as OS treatment. Interestingly, we observed intracellularly vacuole-like PA enrichment in myotube upon the acute tension oscillation, which is distinct from the mainly cytosol and partial plasma membrane-distribution of PABD-GFP when myotube was incubated in isotonic or hypotonic buffers (Figure 1A).

To test whether these PA-enriched vacuoles are derived from the plasma membrane via macropinocytosis, an endocytic process which engulfs large amount of solutes and plasma membranes (Buckley and King, 2017; Yoshida et al., 2018), we added a macropinocytosis-specific cargo, rhodamine-conjugated dextran, into the buffers and found that those PA-enriched vacuoles induced by OS were loaded with rhodamine-dextran (Figure 1B). Furthermore, similar PA-enriched and rhodamine-dextran containing macropinosomes could also be observed in myotubes cultured on silicon membrane that was subjected to 2-min mechanical stretching followed by 2-min relaxation (Figure 1C,D). To confirm the specificity of the PA biosensor, we also examined other lipid markers, Lact-C2-GFP and PLCδ-PH-GFP, to monitor the distribution of phosphatidylserine (PS) and phosphatidyl-4,5-bisphosphate [PI(4,5)P_2_], respectively. Different from the distinct enrichment of PABD-GFP around the rhodamine-dextran positive macropinosome, there was little enrichment of PS or PI(4,5)P_2_ at the macropinosome (Figure 1E). Together, these data suggest that the acute fluctuation of plasma membrane tension leads to the formation of PA-enriched macropinosome in muscle cells.

### PA Is Enriched at the Membrane of Macropinosome but Not Lysosome

**FIGURE 2:**
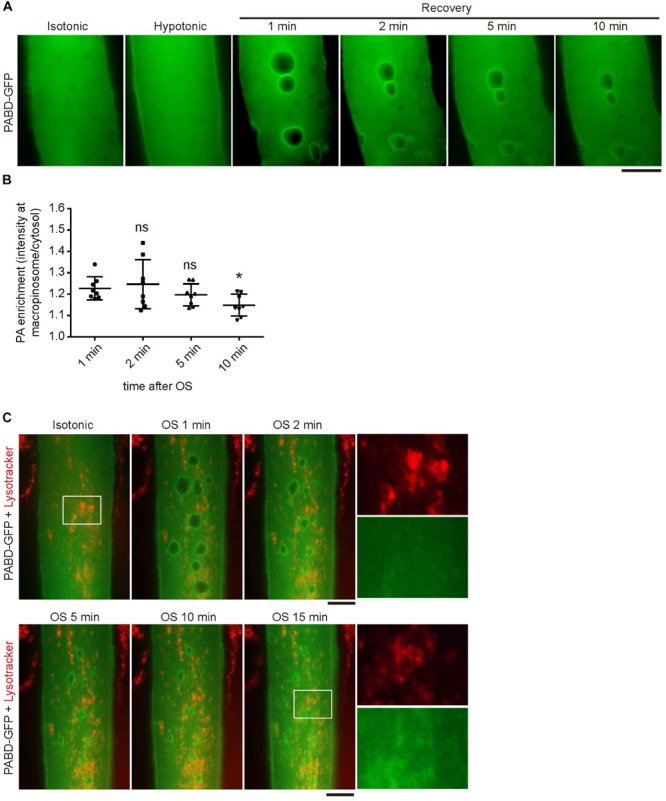
Phosphatidic acid (PA) is enriched at macropinosome but not lysosome upon OS treatment. **(A,B)** The enrichment and duration of PA at macropinosome. PABD-GFP expressing myotube was subjected to OS treatment and time-lapse microscopy images were shown. The intensity of PABD-GFP at the macropinosome or cytosol were quantified upon OS treatment and shown in **(B)**. Eight macropinosomes in **(A)** were quantified and analyzed. **(C)** PABD-GFP was not accumulated on lysosome upon OS treatment. After incubation with lysotracker for 15 min, PABD-GFP expressing myotube was imaged with time-lapse microscopy upon OS treatment. Boxed areas were magnified and shown. Scale bars: 10 mm. ^∗^*p* < 0.05.

Phosphatidic acid is a rapidly metabolized signaling lipid; therefore, we wondered how long PA could remain on macropinosome induced by membrane tension change. By monitoring the signal of PABD-GFP in myotube after OS treatment, we found that the PA-enrichment at macropinosome could last over 10 min upon OS, with a gradual decline of GFP intensity at macropinosome (Figure 2A,B). Furthermore, the tension oscillation-induced PA accumulation could not be observed at the lysosome labeled by lysotracker, indicating the specific enrichment of PA on macropinosome in myotube (Figure 2C).

### PLD2 Localizes at PA-Enriched Macropinosome

**FIGURE 3:**
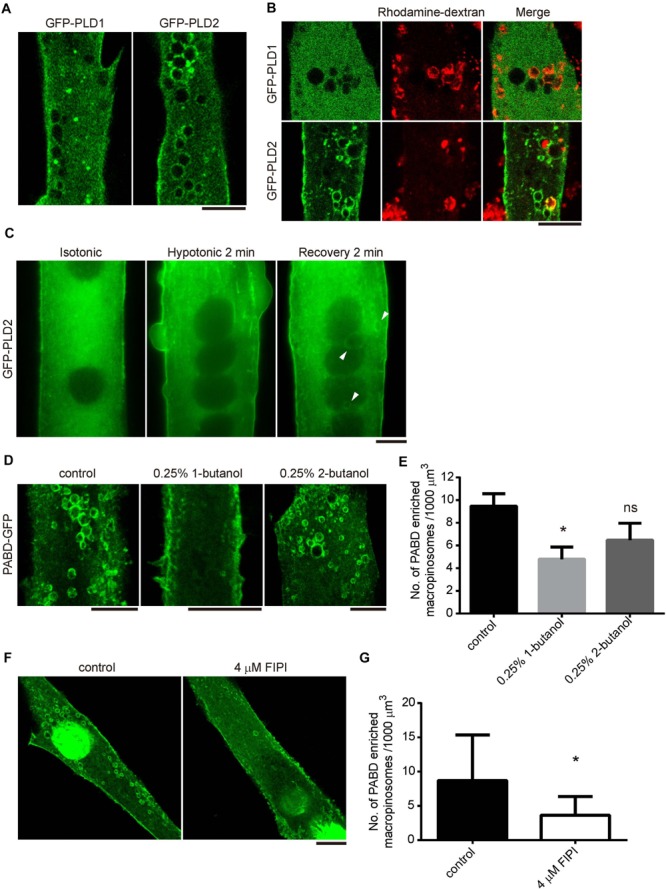
PLD2 is enriched at the tension fluctuation-induced macropinosome. **(A,B)** Distribution of GFP-PLD1 and GFP-PLD2 in myotubes upon OS treatment. GFPP-PLD1 or GFP-PLD2 expressing myotubes were subjected to OS treatment with **(B)** or without **(A)** the presence of rhodamine-dextran. Images were acquired after intensive PBS wash and fixation. Confocal microscopy images were displayed. **(C)** Live cell imagining of GFP-PLD2 in myotube upon OS treatment. Arrowheads indicate GFP-PLD2 at the macropinosome. **(D,E)** The formation of PA-enriched macropinosome was inhibited by PLD inhibitor 1-butanol. PABD-GFP expressing myotubes were pretreated with 0.25% 1- or 2-butanol for 10 min and followed with OS treatment in the presence of indicated butanol **(D)**. The density of PA-rich macropinosome were quantified, compared with control cells and shown in **(E)**. **(F,G)** FIPI inhibited the formation of PA-enriched macropinosome formation. PABD-GFP expressing myotubes were pretreated with vehicle or 4 μM FIPI in 1X PBS for 30 min and followed by OS treatment with or without 4 μM FIPI. The density of PA-positive macropinosome was quantified, compared with control cells and shown in **(G)**. Ten myotubes for each condition were quantified. Data in **(E,G)** were analyzed with one-way ANOVA and Student’s *t*-test, respectively. Scale bars: 10 mm. ^∗^*p* < 0.05.

To understand how PA remained on the surface of macropinosome, we examined the distribution of its producing enzyme, PLD, in myotube upon OS treatment. There are two major isozymes of PLD in skeletal muscle cells, PLD1 and PLD2, which localize differently in many cells (Du et al., 2004; Zeniou-Meyer et al., 2007). Therefore, we examined the distribution of GFP-PLD1 and GFP-PLD2 in C2C12-derived myotubes and observed dramatic accumulation of GFP-PLD2 on the surface of OS-induced macropinosome, while GFP-PLD1 was mainly localized at intracellular vesicles and partially to the plasma membrane (Figure 3A). The enrichment of GFP-PLD2 to the rhodamine-dextran positive macropinosome further suggests that PLD2 is the responsible enzyme for PA production upon tension fluctuation (Figure 3B). We thus took a closer look at the kinetic distribution of GFP-PLD2 in myotube during OS treatment and found it localized mainly at the plasma membrane and cytosol both in isotonic and hypotonic buffers (Figure 3C). However, after 2 min of recovery with isotonic buffer, GFP-PLD2 was shifted to the macropinosome probably along the process of macropinocytosis (white arrow heads, Figure 3C).

To test whether the activity of PLD is critical for the formation of PA-enriched macropinosome, PABD-GFP expressing myotubes were pretreated with the PLD inhibitors, 0.25%1-butanol or 4 μM FIPI for 10 or 30 min respectively, then were subjected to OS treatment. The number of PA-enriched macropinosome was significantly decreased in 1-butanol and FIPI treated myotubes, whereas the control alcohol, 2-butabol, had less effect on tension-induced macropinosome formation (Figure 3D–G and Fang et al., 2001). These results demonstrate that PLD-mediated PA production is responsible for the formation of PA-containing macropinosome in myotube upon tension fluctuation.

### mTOR Signaling Pathway Is Activated by OS Treatment

**FIGURE 4:**
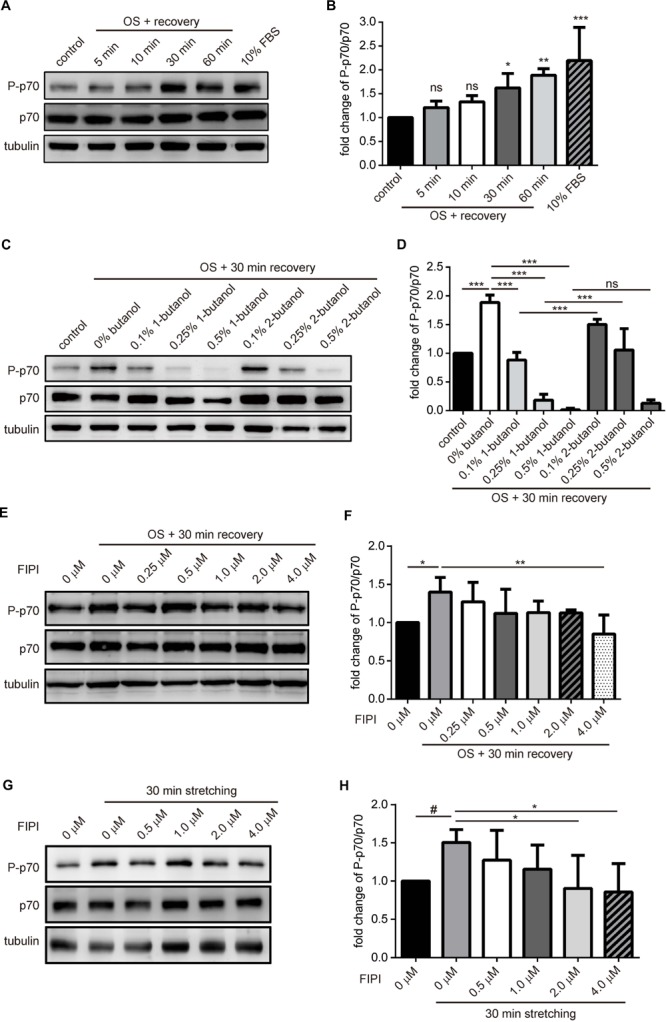
mTOR signaling is activated in myotube upon tension fluctuation. **(A,B)** mTORC1 signaling upon OS treatment. C2C12-derived myotubes were treated with 2-min of 0.25X PBS and followed by indicated period of time for recovery with isotonic DM. Control was myotube incubated with DM without OS treatment whereas 10% FBS sample was myotube treated with 60-min of growth medium. Cell lysates were analyzed with Western blotting to quantify p70 phosphorylation **(A)**. Three independent experiments of the fold change of P-p70/p70 upon OS treatment were quantified with ImageJ and shown as fold change compared with *control cells **(B)**. **(C,D)** 1-Butanol inhibited OS-induced mTOR activation. Myotubes pre-incubated with 10 min of indicated butanol were treated with OS and harvested after 30 min of recovery in DM containing indicative butanol. P-p70 and total p70 were analyzed with Western blotting, quantified with imageJ and shown as fold change compared to no-butanol, OS-treated cells **(D). (E,F)** Dose-dependent effect of FIPI on OS-induced mTOR activation. Myotubes pretreated with 30 min of indicated concentration of FIPI were subjected to OS and harvested after 30 min of recovery in DM with indicative FIPI. P-p70 and total p70 were analyzed with Western blotting, quantified with imageJ and shown as fold change compared to OS-treated cells without FIPI **(F)**. **(G,H)** Dose-dependent effect of FIPI on stretch-induced mTOR activation. Myotubes cultured on silicon membrane were pretreated with 30 min of indicated concentration of FIPI, then subjected to 30-min 0.1 Hz, 20% radial stretching in DM containing indicative FIPI. P-p70 and total p70 were analyzed with Western blotting, quantified with imageJ and shown as fold change compared to the stretched cells without FIPI **(F)**. Three independently-differentiated C2C12 myotubes were used for each experiment, and three sets of data were quantified and analyzed with one-way ANOVA. #*p* < 0.1; ^∗^*p* < 0.05; ^∗∗^*p* < 0.01; ^∗∗∗^*p* < 0.001.*

It has been well-established that PA directly activates mTOR by binding to the FRB domain thus activates its kinase activity *in vitro* and in cell (Yoon et al., 2011b). To know whether the tension fluctuation-induced macropinosome could lead to mTOR activation, we first examined the amount of phosphorylated S6K1/p70, a substrate of mTORC1, in myotubes upon OS treatment. Cell lysates of C2C12-derived myotubes with indicative recovery time upon OS treatment were harvested and immune blotted with anti-phosphorylated p70 (P-p70), anti-p70 and anti-tubulin antibodies. We found that the ratio of P-p70/p70 in myotubes gradually increased upon OS treatment and reached about twofold after 1 h of recovery in DM (Figure 4A,B). Importantly, the OS treatment induced-mTOR activation was blocked by PLD inhibitors, 1-butanol and FIPI, in a dose-dependent manner; whereas the control alcohol 2-butanol had less effect (Figure 4C–F). We further examined this phenomenon in myotubes with cell stretcher and found a similar result that FIPI also inhibits the increase of mTOR activity upon 30-min, 0.1 Hz and 20% radial stretching of myotubes (Figure 4G,H). These results indicate that OS-treatment and mechanical stretch lead to mTOR activation via PLD activity.

### mTOR Is Recruited to PA-Enriched Macropinosome

It was reported that mitogens activate mTOR through PA production at the lysosomal membrane (Yoon et al., 2015). Given the enrichment of PA at macropinosome and the direct interaction between PA and mTOR (Figure 1B and Fang et al., 2001; Yoon et al., 2011b), we wondered if the tension fluctuation-induced macropinosome could serve as a platform to recruit mTOR. Thus we used immunofluorescence staining to visualize the distribution of endogenous mTOR in PABD-GFP expressing myotube before and after OS treatment.

**FIGURE 5:**
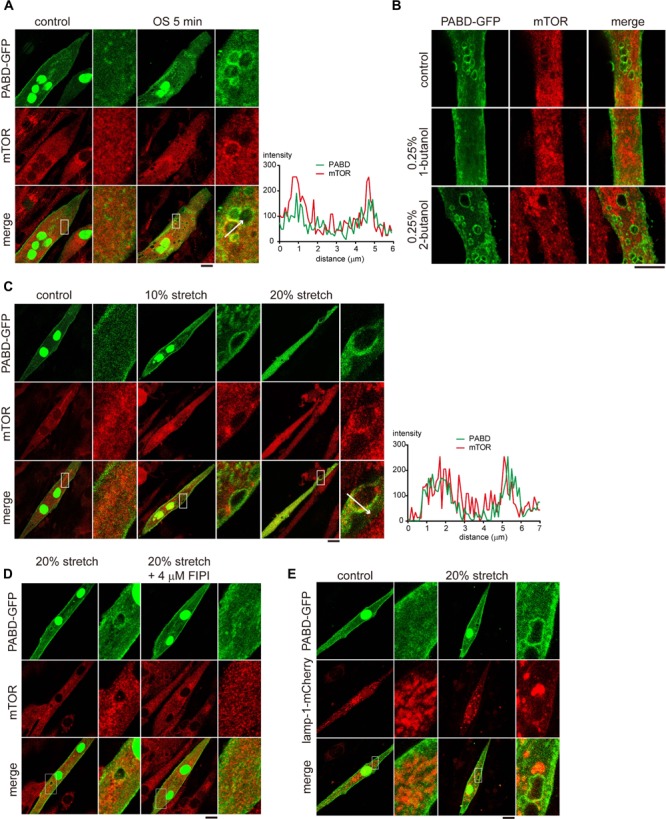
mTOR is localized to the PA-enriched macropinosome. **(A)** Localization of mTOR in OS-stimulated myotube. PABD-GFP expressing myotubes with or without OS treatment, were immunofluorescence stained to label the endogenous mTOR and were imaged with confocal microscopy. Boxed areas were magnified and shown on the right panel. The fluorescence intensity profiles from the line scan are shown to illustrate the co-localization of mTOR and PABD-GFP. **(B)** 1-Butanol inhibits the targeting of mTOR to macropinosome upon OS treatment. PABD-GFP expressing myotubes with 0.25% 1- or 2-butanol pre-incubation were subjected to OS treatment and further stained for endogenous mTOR distribution. **(C)** Single cycle of mechanical stretching and relaxation leads to mTOR targeting to PA-rich macropinosome. PABD-GFP expressing myotubes cultured on silicon membrane were subjected to 0, 10, or 20% radial stretch for 2 min and followed by 2-min resting. After immunofluorescence staining of endogenous mTOR, cells were imaged with confocal microscopy. **(D)** FIPI inhibits the formation of mTOR- and PA-enriched macropinosome upon stretching. PABD-GFP expressing myotubes were pre-incubated with DMSO or FIPI and then subjected to single cycle of mechanical stretch and relaxation. After fixation and immunofluorescence staining, the distribution of PABD-GFP and endogenous mTOR were imaged with confocal microsocpy. **(E)** Mechanical stimulation-induced PA is not enriched at lysosome. After single cycle of 20% stretch, PABD-GFP and Lamp1-mCherry expressing myotubes were fixed and imaged with confocal microscopy. Scale bars: 10 μm.

In contrast to the mainly cytosolic distribution of mTOR in myotube cultured in isotonic buffer, mTOR was partially enriched at the PA-containing macropinosome after OS treatment (Figure 5A). The fluorescent intensity profiles from the line scan further supports the co-enrichment of PA and mTOR in myotube upon OS-treatment (Figure 5A). Consistent with previous observation that 1-butanol resulted in a reduction of PA-enriched macropinosome (Figure 3D), there was no mTOR positive macropinosome in 1-butanol treated myotube upon OS treatment (Figure 5B). Lastly, to confirm that macropinosome could function as a platform to recruit mTOR in muscle upon mechanical stretch, PABD-GFP expressing myotube was cultured on silicon membrane and subjected to 2-min stretching, 2-min relaxation and followed by fixation and immunofluorescence staining for mTOR localization. In line with the results in OS-treated myotubes, we observed co-localization of PABD-GFP and mTOR at macropinosome in myotube after both 10 or 20% radial stretching and resting (Figure 5C). Importantly, the formation of PA and mTOR-enriched macropinosome upon mechanical stretch were inhibited by PLD inhibitor, FIPI (Figure 5D). Furthermore, the PA and mTOR-enriched macropinosome resulted from stretching was devoid of the lysosomal protein, Lamp1-mCherry (Figure 5E). Together, these results suggest that tension fluctuation-induced macropinosome functions as a platform for mTOR recruitment and activation via PLD activity.

## Discussion

**FIGURE 6:**
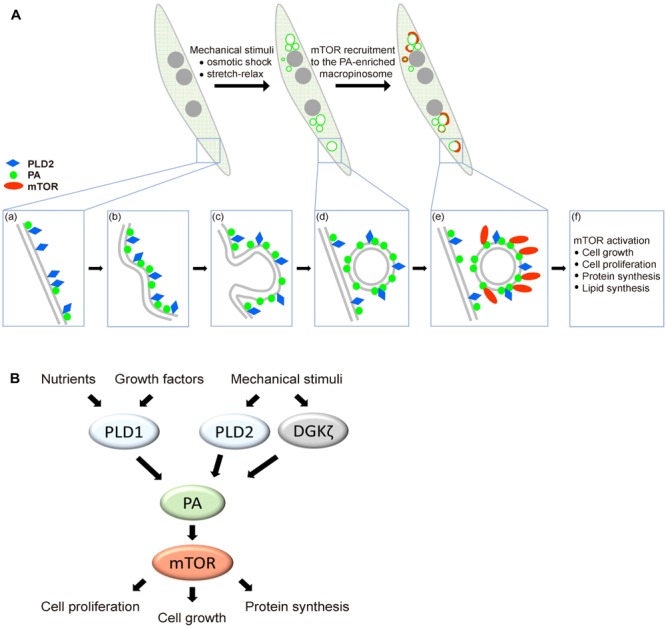
Hypothetical model of mechanical stimulation-induced mTOR distribution and activation in muscle. **(A)** Mechanical stimulation leads to the formation of PA-enriched macropinosome which serves as a platform for mTOR recruitment. Plasma membrane-localized PLD2 **(a)** is activated via mechanical stimulation and the production of PA is involved in macropinosome formation **(b–d)**. mTOR is recruited to the PA-enriched macropinosome and results in mTOR signaling activation **(e,f)**. **(B)** Biochemical and mechanical transduction pathway for mTOR activation. Growth factors stimulate the activation of mTOR mainly through the activity of PLD1 on lysosome, whereas mechanical stimulation induces mTOR activation and localization to macropinosome mainly through PLD2.

Numerous studies in different organisms have discovered that localization of TOR is tightly regulated to enact precise spatial and temporal control of cell growth (Laplante and Sabatini, 2009; Betz and Hall, 2013). Distinct from the lysosomal localization of mTORC1 upon mitogenic stimulation, here we discovered that mechanical stimulation induces the targeting of mTOR to PA-enriched macropinosome as well as the activation of mTORC1 signaling in muscle cell. Interestingly, this mechanical stimulated PA-containing macropinosome was associated with PLD2, yet much less with PLD1 (Figure 6A). Together, we hypothesize that biochemical stimuli activate mTORC1 at lysosomal membrane mainly through PLD1, whereas mechanical stimulation activates and recruits mTORC1 to the macropinosome via PLD2 activity (Figure 6B).

Mechanical loads have been known to promote muscle mass accumulation via PLD- and PA-mediated mTOR activation (Hornberger et al., 2006; Foster et al., 2014). However, the molecular mechanism of PLD activation by mechanical stretch remains unclear. Here, we observed that the fluctuation of plasma membrane tension leads to the accumulation of PA on macropinosome and mTOR activation, indicating that membrane tension is probably the underpinning of PLD2 activation upon mechanical stretch and relaxation. In line with this hypothesis, Petersen et al. recently reported that PLD2 could be activated by mechanical stimulation, e.g., shearing force, which disrupts lipid raft thus results in the mixing of PLD2 and its substrate phosphatidylcholine on the plasma membrane (Petersen et al., 2016). Therefore, the stretching and relaxation of muscle cell may activate PLD2 with a similar mechanism and lead to PA production. After the cycle of stretching and relaxation, the flaccid plasma membrane which was due to caveolae disassembly (Sinha et al., 2011; Lo et al., 2015), were restored by macropinocytosis where PLD2 was also internalized (Figure 6A). With the presence of PLD2, mechanical stimulation-induced macropinosome could function as a platform to recruit and activate mTOR via PA. Notably, 2 min of OS treatment could lead to mTOR activation for about 1 h (Figure 4B).

Phosphatidic acid is a well-appreciated signaling lipid that directly activates mTOR via binding to the FRB domain, competing with the inhibitory proteins, and allosterically activating mTORC1 (Yoon et al., 2011b, 2015). However, how mTORC1 could be activated by PA at the macropinosome, a structure that would presumably lack lysosomal proteins required for classical mTORC1 activation? Given that Rags are responsible for the recruitment of mTORC1 to the lysosome where Rheb activates mTORC1 by binding and allosterically accelerating the catalysis (Betz and Hall, 2013; Yang et al., 2017), we expect that high density of PA at the macropinosome may be sufficient for the recruitment and activation of mTORC1 even in the absence of Rags and Rheb.

While PLD1 is the major PA-generating enzyme responsible for mitogen-stimulated mTOR activation (Fang et al., 2001; Foster et al., 2014; Yoon et al., 2015), here we find that PLD2 is the major isoform associated with tension fluctuation-induced macropinosome, indicating a functional specificity of PLD1 and PLD2 for biochemical and mechanical activation of mTOR in muscle cells, respectively (Figure 6B). However, it has also been reported that another PA producing enzyme diacylglycerol kinase ζ (DGKζ), was also involved in the PA-mediated mTOR activation in mechanically stimulated muscle cells (You et al., 2014). Therefore, we speculate that multiple PA-generating enzymes may contribute to the response of muscle cell toward mechanical stimuli, leading to PA production and mTOR activation probably at different membrane compartments. The different subcellular localization of DGK and PLD, as well as the different substrates of these enzymes, diacylglycerol versus phosphatidylcholine respectively, may equip them as distinct sensors of different mechanical/biochemical stimulations.

In this study, we discovered that mechanical stimulated-mTOR activation takes place at the PLD2-associated macropinosome which serves as a platform for mTOR targeting and activation via the enriched PA. Our findings thus pave the way for further studies to understand the detailed signaling compartmentalization of mTOR activation upon mechanical loading. There are still many unanswered questions need to be addressed, e.g., Are Rag and Rheb small GTPases dispensable for mTOR activation on macropinosome? Does mTOR phosphorylate the same set of substrates where it is activated on lysosome or macropinosome? A comprehensive understanding of the spatial, temporal, and dynamic activation of mTOR signaling is required, and the answers of them will bring significant insights into the mTOR-mediated development and disease progression.

## Author Contributions

S-SL and Y-WL designed and performed experiments as well as wrote the manuscript.

## Conflict of Interest Statement

The authors declare that the research was conducted in the absence of any commercial or financial relationships that could be construed as a potential conflict of interest.
